# Adiposity, Obesity, and Arterial Aging: Longitudinal Study of Aortic Stiffness in the Whitehall II Cohort

**DOI:** 10.1161/HYPERTENSIONAHA.115.05494

**Published:** 2015-07-08

**Authors:** Eric J. Brunner, Martin J. Shipley, Sara Ahmadi-Abhari, Adam G. Tabak, Carmel M. McEniery, Ian B. Wilkinson, Michael G. Marmot, Archana Singh-Manoux, Mika Kivimaki

**Affiliations:** From the UCL Research Department of Epidemiology and Public Health, London, United Kingdom (E.J.B., M.J.S., S.A.-A., A.G.T., C.M.M., M.G.M., A.S.-M., M.K.); Semmelweis University Faculty of Medicine, 1st Department of Medicine, Budapest, Hungary (A.G.T.); Clinical Pharmacology Unit, Division of Experimental Medicine and Immunotherapeutics, University of Cambridge, Cambridge, United Kingdom (C.M.M., J.B.W.); INSERM, Centre for Research in Epidemiology & Public Health, Hôpital Paul Brousse, Bâtiment, France (A.S.-M.).

**Keywords:** aging, arterial stiffness, epidemiology, obesity, longitudinal studies

## Abstract

We sought to determine whether adiposity in later midlife is an independent predictor of accelerated stiffening of the aorta. Whitehall II study participants (3789 men; 1383 women) underwent carotid-femoral applanation tonometry at the mean age of 66 and again 4 years later. General adiposity by body mass index, central adiposity by waist circumference and waist:hip ratio, and fat mass percent by body impedance were assessed 5 years before and at baseline. In linear mixed models adjusted for age, sex, ethnicity, and mean arterial pressure, all adiposity measures were associated with aortic stiffening measured as increase in pulse wave velocity (PWV) between baseline and follow-up. The associations were similar in the metabolically healthy and unhealthy, according to Adult Treatment Panel-III criteria excluding waist circumference. C-reactive protein and interleukin-6 levels accounted for part of the longitudinal association between adiposity and PWV change. Adjusting for chronic disease, antihypertensive medication and risk factors, standardized effects of general and central adiposity and fat mass percent on PWV increase (m/s) were similar (0.14, 95% confidence interval: 0.05–0.24, *P*=0.003; 0.17, 0.08–0.27, *P*<0.001; 0.14, 0.05–0.22, *P*=0.002, respectively). Previous adiposity was associated with aortic stiffening independent of change in adiposity, glycaemia, and lipid levels across PWV assessments. We estimated that the body mass index–linked PWV increase will account for 12% of the projected increase in cardiovascular risk because of high body mass index. General and central adiposity in later midlife were strong independent predictors of aortic stiffening. Our findings suggest that adiposity is an important and potentially modifiable determinant of arterial aging.

## Abstract 2

Supplemental Digital Content is available in the text.

**See Editorial Commentary, pp 270–272**

Aortic stiffness is a key characteristic of arterial aging and a strong predictor of cardiovascular events.^1–4^ An exaggerated blood pressure response during daily activities, producing increased load on the heart and blood vessels, is among the pathogenic consequences of an inelastic aorta.^5^ In view of the important pathophysiological role of aortic stiffness, the modifiable determinants of stiffening need to be better understood by studying longitudinal changes. As a consequence of the obesity epidemic, general and central adiposity are highly prevalent. Both are associated with aortic stiffness^6–9^ and increased risk of cardiovascular disease.^10,11^ Sparse longitudinal evidence to date leaves uncertainty about the role of adiposity in later midlife in progression of aortic stiffness.^12–15^

In this context, we assessed the effect of adiposity on aortic stiffening in a large longitudinal study with 2 measurements of carotid-femoral pulse wave velocity (PWV) 4 years apart, using applanation tonometry. We compared prospective effects of differing measures: general adiposity by body mass index (BMI), central adiposity by waist circumference and waist:hip ratio, and fat mass percent by body impedance. We considered the effect of concurrent change in adiposity. After stratifying the cohort into metabolically healthy and unhealthy participants, defined according to National Cholesterol Education Program Adult Treatment Panel-III criteria, excluding waist circumference, we examined the effects of adiposity on aortic stiffening.^16^ We examined the biological pathways linking adiposity with aortic stiffening in a series of models controlling for chronic disease, use of medication, heart rate, metabolic risk factors, and markers of inflammation.

## Methods

### Study Population and Design

The Whitehall II study is a longitudinal study of 10 308 male and female civil servants (initially aged 35–55 years) recruited in 1985 to 1988 on the basis of staff lists from offices located in central London. The response rate was 73%. Details of the cohort investigations and loss to follow-up have been published.^17^ The cohort has been followed with clinical examinations every 4 to 5 years. Research Ethics Committee approval and written informed consent from each participant were obtained at each study phase. The present sample included those who had a PWV measurement at the 2008 to 2009 (n=4347) or 2012 to 2013 (n=4344) assessments, screened at our clinic or at home (20% of participants) using the same protocol. After exclusion of 65 PWV observations with missing covariates, the analyses used 8636 measurements from 5172 participants, of whom 3454 provided PWV measurements on both occasions.

### Measures

Aortic PWV was assessed between carotid and femoral sites using applanation tonometry (SphygmoCor; Atcor Medical, Australia). We used standard protocols to diagnose vascular disease and diabetes mellitus and to assess antihypertensive medication use. Risk factors were measured in 2003 to 2004 and 2008 to 2009 to provide mean exposure in the 5 years before baseline PWV assessment in 2008 to 2009. Description of measurement protocols is available in the online-only Data Supplement.

### Statistical Analysis

Distributions of adiposity measures were categorized in sex-specific thirds and standardized units. Linear mixed models were used to estimate the relationship of adiposity with PWV in 2008 to 2009 and change in PWV between 2008 to 2009 and 2012 to 2013. These models use all available PWV data and account for correlation between repeated measures within individuals. We fitted the intercept and slope with time as random effects for individual differences in PWV at baseline and rate of change over follow-up. The main effect for adiposity estimates the effect on PWV at baseline (2008–2009), whereas the adiposity×time interaction term estimates the effect of adiposity on change in PWV between 2008 to 2009 and 2012 to 2013 as a 5-year change.

Models were adjusted for age, sex, ethnic group, and mean arterial pressure at the time of PWV measurement. Change in PWV by third of each adiposity measure and per 1SD increment in adiposity was estimated. PWV changes were estimated separately among those who were metabolically healthy and unhealthy according to ATP-III criteria excluding waist circumference.^16^ Further models additionally adjusted for (1) chronic disease and antihypertensive medication, (2) heart rate, (3) serum triglycerides, high-density lipoprotein, fasting glucose, and hemoglobin A1c, (4) C-reactive protein and interleukin-6, and (5) all factors. Main analyses using logarithmically transformed PWV, in place of PWV, produced similar strength of associations with adiposity. We conducted a sensitivity analysis to examine potential bias arising from change in central adiposity over the follow-up period.^18^ We calculated path length at follow-up adjusted for the association between change in waist circumference and change in measured aortic path length. Adjustment was based on a sex-specific regression model of change in path length against change in waist circumference. The path length was adjusted to remove the effect of change in waist circumference on path length in each individual. We used data from a published meta-analysis^4^ to estimate change in cardiovascular disease risk resulting from increased log PWV because of a 1SD higher BMI and expressed this change according to Wormser et al^11^ to estimate the proportion of this increase mediated through PWV.

## Results

The mean age of participants was 65.5 years, and they were predominantly of white ethnic origin. Fewer than 20% had prevalent chronic disease, and around one third were taking antihypertensive medication. Mean BMI was 26.5 kg/m^2^. Detailed characteristics of the sample at baseline are shown in Table S1 in the online-only Data Supplement.

**Figure 1.**
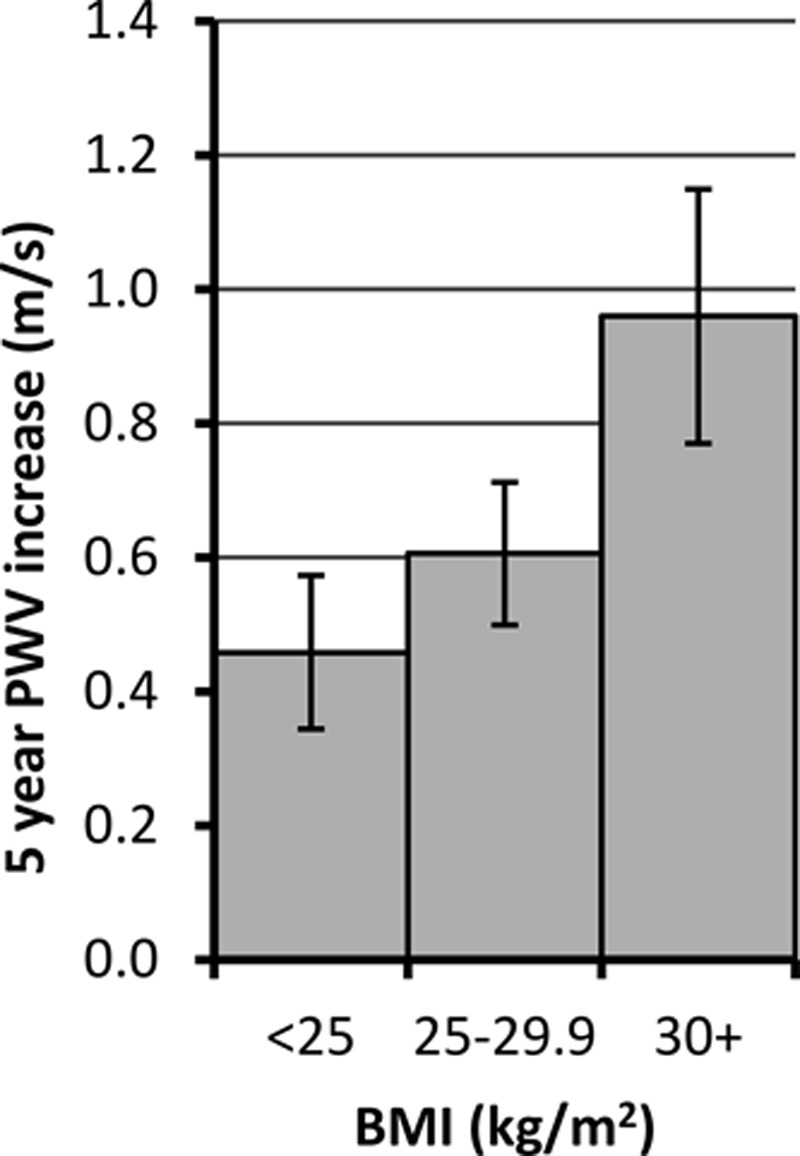
Pulse wave velocity (PWV) change per 5 years (m/s) from age 60 by World Health Organization body mass index (BMI) groups in men and women; 8661 person-observations. Estimates adjusted for age, sex, ethnicity, and mean arterial pressure. *P* value for trend across groups is <0.001. *P* value for departure from linear trend is 0.16. Underweight/normal weight, n=2065; overweight, n=2268; and obese, n=856.

Age, ethnicity, and disease status were associated with baseline PWV after adjustment for mean arterial pressure (Table S2). Older age, disease status, and antihypertensive medication use were associated with greater increases in PWV over follow-up. In analyses adjusted for covariates (Table S3), the mean difference in baseline PWV expressed as proportion of SD of PWV in 2008 to 2009 across each third of mean BMI, waist circumference, waist:hip ratio, and fat mass percent was 11%, 16%, 18%, and 13%, respectively. There was a linear association between each measure of adiposity and PWV change (all *P*<0.001). The size of increase in PWV was strongly related to World Health Organization BMI categories (Figure 1).

**Figure 2.**
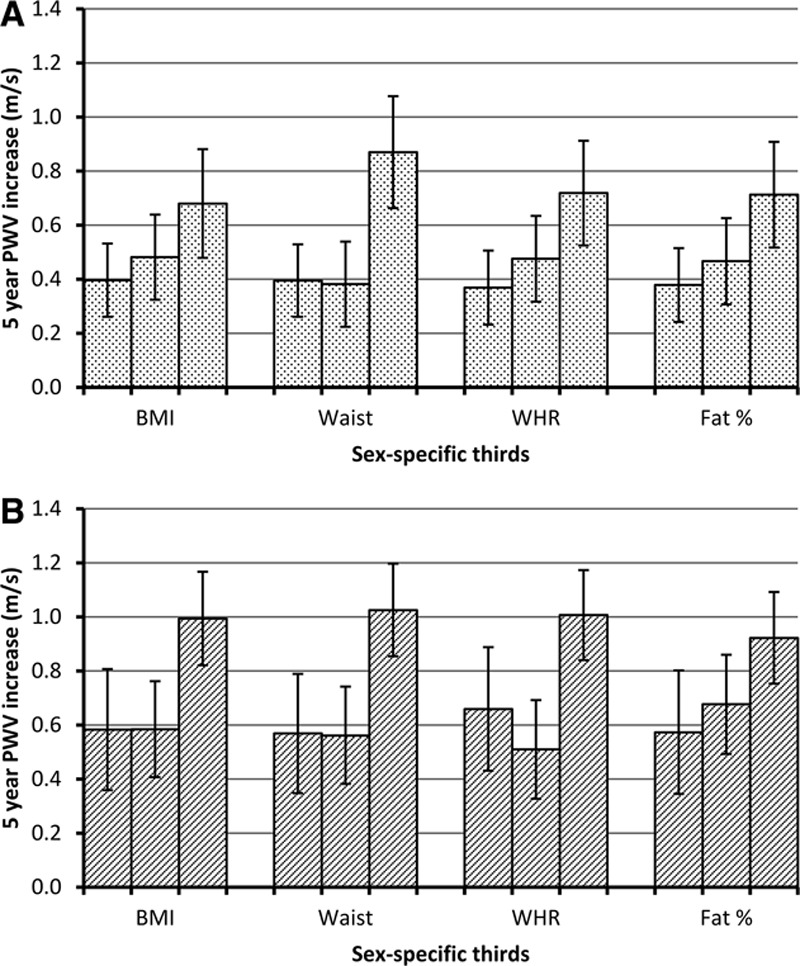
Pulse wave velocity (PWV) change per 5 years (m/s) from age 60 by thirds of adiposity in men and women among metabolically healthy (**A**) and unhealthy (**B**) participants (Adult Treatment Panel-III definition excluding waist circumference). Estimates adjusted for age, sex, ethnicity, and mean arterial pressure. *P* values for trend and departure from trend for metabolically healthy: body mass index (BMI), 0.02 and 0.56; waist, <0.001 and 0.01; waist:hip ratio (WHR), 0.03 and 0.48; fat percent, 0.02 and 0.56 and for metabolically unhealthy: BMI, 0.001 and 0.07; waist, <0.001 and 0.04; WHR, 0.002 and 0.005; fat percent, 0.009 and 0.54.

The influence of risk factor status on longitudinal change in PWV according to the degree of adiposity was examined in metabolically healthy and unhealthy participants, defined using the Adult Treatment Panel-III definition, excluding waist circumference (Figure 2). There was a significant 5-year increase in PWV according to all adiposity measures, based on tests for linear trend, with similar trends in the metabolically unhealthy and healthy groups. There was evidence of departure from linearity for the longitudinal effect of waist circumference.

Table shows the cross-sectional difference and change in PWV associated with adiposity measures after controlling separately for disease status and antihypertensive medication, heart rate, metabolic factors, inflammatory markers, and all of the foregoing. The standardized effect of each adiposity measure on PWV change over the follow-up in the maximally adjusted model was similar in size and was reduced by one quarter to one third compared with the minimally adjusted model (model 1). Sensitivity analysis with additional adjustment for the association between change in waist circumference and change in measured aortic path length between baseline and follow-up confirmed the stability of our findings (Table S4). Minimally adjusted models using baseline path length to calculate the follow-up PWV again confirmed our findings (Table S5).

**Table.**
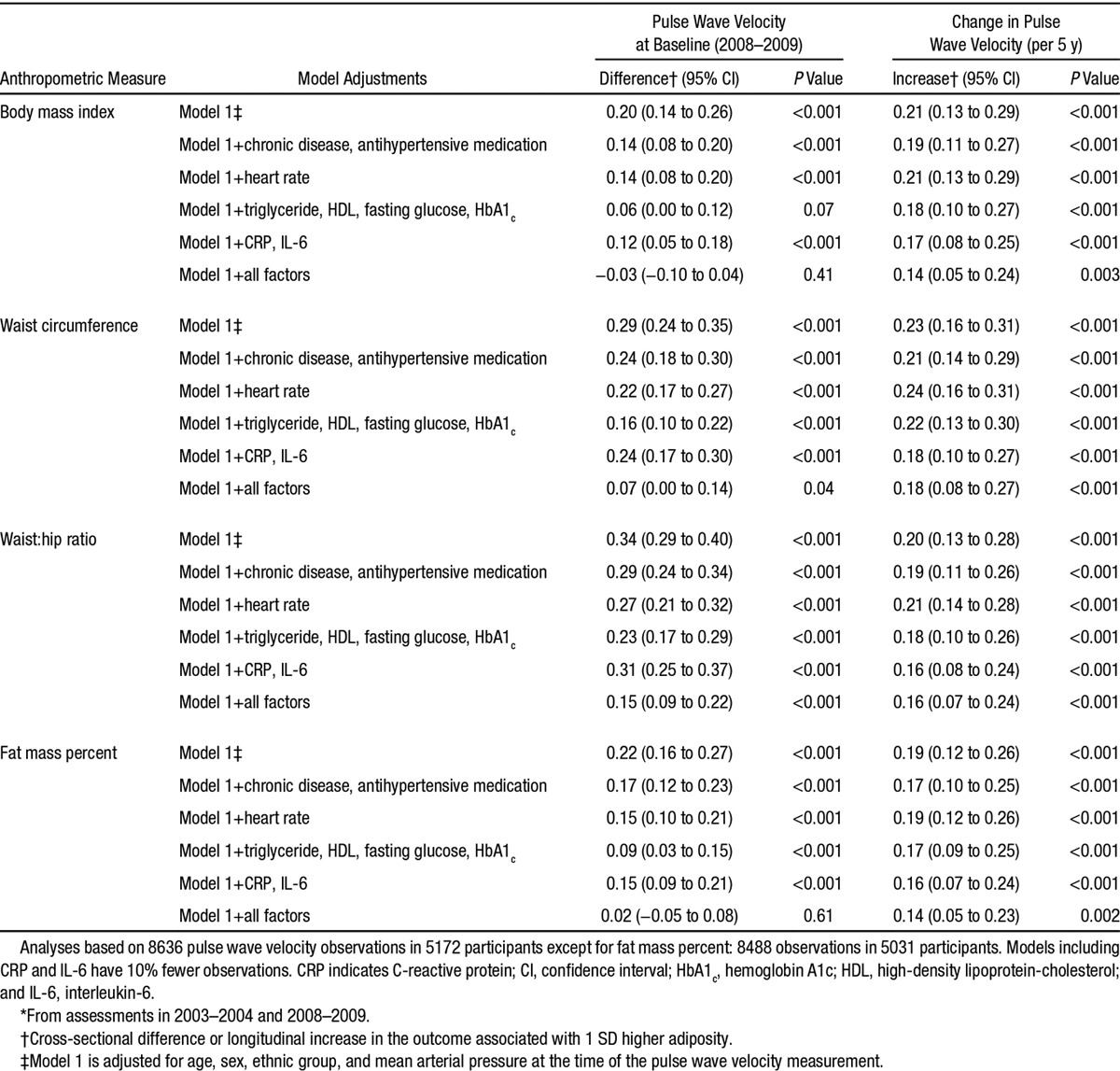
Association of Mean* Anthropometric Measures With Pulse Wave Velocity at Baseline (2008–2009) and 5-y Change in Pulse Wave Velocity

The effects of previous adiposity, based on the 4 measures, were not confounded by changes in adiposity across (concurrent with) the PWV assessments or by changes in available risk factors (triglyceride, high-density lipoprotein, fasting glucose, hemoglobin A1c, and heart rate; Table S6). Effect modification because of degree of change in adiposity across PWV observations was not evident when observations were stratified at respective medians (Table S7). An effect of previous adiposity level on increase in PWV was observed in all subgroups. The effect of previous BMI, waist circumference, and fat mass percent on PWV change was larger in hypertensive than normotensive participants (Table S8). There was no effect modification according to sex or diabetic status.

Based on meta-analytic findings, 12% of the projected increment in cardiovascular disease risk resulting from higher BMI was because of increased PWV.

## Discussion

### Principal Findings

This large longitudinal study of men and women shows that adiposity in later midlife predicts accelerated progression of aortic stiffness. Progression was based on 2 assessments, in 2008 to 2009 and 2012 to 2013, by carotid-femoral applanation tonometry using a standard protocol. The stiffening effect of adiposity was similar in those defined as metabolically healthy and metabolically unhealthy according to Adult Treatment Panel-III criteria. Stiffening was independent of change in adiposity, glycemia, and lipid levels between PWV assessments. Taking account of chronic disease status, use of medication, heart rate, metabolic health, and markers of inflammation, the robust association with increase in PWV over the follow-up suggests that adiposity is an important risk factor for aortic stiffening. Our novel longitudinal findings have clinical and public health significance for older people.

We studied 4 noninvasive measures of adiposity. Observed associations with PWV increase were similar whichever measure was used. Tests for nonlinearity suggested that increase in PWV during the follow-up was linked with high rather than intermediate levels of central adiposity. Some cross-sectional studies^6,7,9,19^ have identified central or visceral adiposity as more strongly correlated than general adiposity with PWV. A longitudinal study showed that clustering of metabolic risk factors was associated with PWV increase.^13^ The present larger study shows that progression tended to be faster (men: 0.25 m/s per 5 years; *P*=0.002 and women: 0.36 m/s per 5 years; *P*=0.02) in those who were metabolically unhealthy regardless of degree of adiposity. Nonetheless, all adiposity measures were robust predictors of accelerated aortic stiffening after adjustment for metabolic and inflammatory risk factors.

### Research in Context

The determinants of aortic stiffening have been under active investigation for 3 decades. A cross-sectional study in the 1980s in China identified urbanicity, high salt intake, and hypertension, as well as age, as risk factors for aortic stiffness.^1^ The association between prevalence of hypertension and arterial stiffness within this population was accompanied by low prevalence of atherosclerosis and low serum cholesterol levels. Since then, studies in general population samples have found associations between PWV and lipid, glycemic, and inflammatory risk factors,^8,20,21^ as well as with obesity.^6,7,9,19^ These cross-sectional findings are inconsistent and temporality could not be examined.

The hypothesis that hypertension is the primary factor and aortic stiffness is secondary^1,8^ is challenged by recent longitudinal studies of disease-free nonhypertensive older adults. These data show that an inelastic aorta predicts incident hypertension and the degree of subsequent blood pressure rise.^14,22,23^ Notably, in the Framingham Offspring Study, high PWV predicted incident hypertension, but initial blood pressure did not predict PWV at follow-up.^14^ Because there may be a 2-way association between blood pressure and PWV,^15^ we controlled for mean arterial pressure, to control for aortic distension during PWV determination but not for systolic, diastolic, or pulse pressure to avoid overadjustment. In this study, previous adiposity was associated with a larger increase in PWV in hypertensive than in normotensive participants.

### Implications

Aortic stiffness is an independent risk factor for incident hypertension, cardiovascular disease, and stroke,^4,23^ and it is associated with age-related physical^2^ and cognitive functioning in older people.^24^ In addition to the known effects of raised BMI on cardiovascular disease risk,^11,25^ our prospective longitudinal findings, based on >5000 participants, add to the evidence that excess adiposity and class I obesity are important determinants of the rate of arterial aging. On the basis of pooled analyses of incident cardiovascular disease, we estimate that 12% of the projected increase in cardiovascular disease because of raised BMI may be attributable to arterial stiffness in this age group.^4,11^ The adiposity–arterial stiffening pathway may be a key target for efforts to compress morbidity and postpone functional decline.^26,27^

Behavior change and pharmacological treatments are relevant potential interventions. Weight loss may lead to a useful reduction in PWV.^28,29^ Exercise capacity and aortic stiffness are inversely associated.^30^ Short-term trials suggest that moderate aerobic exercise in midlife (fast walking, jogging, and swimming) reduces aortic PWV.^31^ Paradoxically, regular resistance exercise (free weights and weight training machines) may increase PWV.^32,33^ Reduction of adiposity-related risk factors with statin therapy^34^ may be effective in lowering PWV; however, the evidence is inconclusive.^35^ Antihypertensive drugs of various classes, including angiotensin-converting enzyme inhibitors, may prove valuable for lowering PWV in overweight and obese individuals.^36,37^

### Mechanisms

The effects of adiposity in the 5 years before the baseline PWV assessment on aortic stiffening over the follow-up were little confounded by changes in adiposity and risk factor levels across PWV assessments. As in a cross-sectional study, there were substantial reductions in the maximally adjusted associations of PWV with BMI and fat mass percent at baseline because of risk factor clustering. In contrast, there were small attenuations in the longitudinal part of the model after these adjustments. The longitudinal findings suggest that metabolic and inflammatory processes contribute to the effects of adiposity on aortic stiffening. Higher adiposity is associated with higher heart rate, but this source of mechanical stress on the aortic wall did not seem to contribute to the pathophysiologic process.^38^ Potential explanations for the unexplained longitudinal effect include a vicious circle of high adiposity, exercise intolerance, arterial stiffening, and physical inactivity,^5^ unmeasured pathways, such as that involving endothelial regulation^39^ and adipokines,^40^ and inadequate measurement of pathways represented in the maximally adjusted model.

### Strengths and Weaknesses

This is the largest longitudinal study of adiposity and aortic PWV to date. We used the same gold-standard tonometry method at baseline and follow-up.^41^ At each contact phase, the average of 2 PWV measurements was used, with a third measurement used when, rarely, the difference between the first 2 was >0.5 m/s. This rigorous protocol contributed to the power of the study to detect change in PWV over the modest interval between measurement occasions. Determination of path length with a tape measure is the weakest element of the protocol because estimation of PWV involves a degree of systematic bias with respect to waist circumference.^18^ We found that there was an association between the modest change in waist circumference (SD, 5.2 cm) and change in the aortic path length over the follow-up period. However, our sensitivity analysis correcting for this bias showed that it did not distort the findings. Further analysis using path length at baseline to calculate both first and second PWV measurements circumvents the problem of change in adiposity and provides strong evidence that our longitudinal findings are robust. Separately, additional model adjustment for body height did not change the results (data not shown).

PWV was first measured in the cohort when the youngest participants were aged 55. We therefore cannot contribute to the equivocal evidence concerning adiposity and PWV in younger adults.^7,42^ Vascular risk factor levels tend to be lower in our health-conscious occupational cohort than in the general population; however, systematic comparison of risk factor effects on coronary risk between Whitehall II, Framingham, and the British Regional Heart Study shows that our etiologic estimates are generalizable.^43^

### Perspectives

Aortic stiffness predicts cardiovascular events over and above standard risk factors. A high age-specific level of aortic stiffness is linked with poor aging-associated physical function, for example, slow-walk speed, poor lung function, and functional limitations. In this context, we measured carotid-femoral PWV on 2 occasions 4 years apart to show that degree of adiposity is a robust predictor of aortic stiffening. Adiposity was measured noninvasively in several ways (BMI, waist circumference, waist:hip ratio, and fat mass percent by body impedance) in the 5 years before baseline PWV assessment. Each adiposity measure predicted change in aortic PWV. Serial assessments of aortic stiffness may prove to be a valuable tool for monitoring vascular aging and tracking the effect of interventions to slow or reverse stiffening of the aorta.

### Conclusions

Adiposity was a robust predictor of aortic stiffening in the presence and absence of co-occurring metabolic risk factors and inflammation. General and central obesity and fat mass percent were equally predictive of aortic stiffening in our study sample. Adiposity and associated metabolic risk factors are modifiable, suggesting that the adverse health and functional consequences of aortic stiffening may be postponed or prevented.

## Acknowledgments

We thank all of the participating civil service departments and their welfare personnel and establishment officers; the Occupational Health and Safety Agency; the Council of Civil Service Unions; all of the participating civil servants in the Whitehall II Study; and all members of the Whitehall II Study team. The Whitehall II study team is composed of research scientists, statisticians, study coordinators, nurses, data managers, administrative assistants, and data entry staff, who make the study possible.

## Sources of Funding

This work was supported by the British Heart Foundation (RG/13/2/30098), British Medical Research Council (K013351), the British Health and Safety Executive, the British Department of Health, the British Stroke Association (TSA 2008/05), the US National Heart, Lung, and Blood Institute (R01HL036310), and the US National Institute on Aging (R01AG013196 and R01AG034454). M. Kivimaki was supported by a professorial fellowship from the Economic and Social Research Council. I.B. Wilkinson is a British Heart Foundation senior fellow. C.M. McEniery and I.B. Wilkinson received support from the Cambridge National Institute for Health Research Biomedical Research Centre.

## Disclosures

None.

## Supplementary Material

## Novelty and Significance

### What Is New?

- We collected 2 measures of carotid-femoral pulse wave velocity 4 years apart starting at the mean age of 66 years in a cohort of 5172 men and women.
- We showed that the degree of adiposity in the 5 years before baseline assessment, whether measured as general obesity, central obesity, or fat mass percent, was a robust predictor of aortic stiffening.
- Adiposity-associated stiffening of the aorta was observed in those who were metabolically healthy, as well as those who were not.

### What Is Relevant?

- The longitudinal relationship between aortic stiffening and hypertension remains unclear.
- Longitudinal analysis from the Framingham Offspring study suggests that aortic stiffness may be a precursor of hypertension in older people.

### Summary

Adiposity may be an important and potentially modifiable determinant of aortic stiffness and of incident hypertension in older people.
