# Supplementary material for: Adiposity, Obesity, and Arterial Aging: Longitudinal Study of Aortic Stiffness in the Whitehall II Cohort
Source: Hypertension. 2015 Jul 8;66(2):294–300. doi: 10.1161/HYPERTENSIONAHA.115.05494 (PMC4490910; doi:10.1161/HYPERTENSIONAHA.115.05494)
Supplement: Supplementary file 1 [file hyp-66-294-s001.docx]

Data Supplement

**DIPOSITY, OBESITY AND ARTERIAL AGING: LONGITUDINAL STUDY OF AORTIC STIFFNESS IN THE WHITEHALL II COHORT**

Eric J Brunner PhD FFPH^a^, Martin J Shipley MSc^a^, Sara Ahmadi-Abhari MD PhD^a^, Adam G Tabak MD^a,b^, Carmel M McEniery PhD^c^, Ian B Wilkinson MBBS^c^, Michael G Marmot FRCP^a^, Archana Singh-Manoux PhD^a,d^, Mika Kivimaki PhD^a^

^a^UCL Research Department of Epidemiology and Public Health, London, UK

^b^Semmelweis University Faculty of Medicine, 1^st^ Department of Medicine, Budapest, Hungary ^c^Clinical Pharmacology Unit, Division of Experimental Medicine and Immunotherapeutics, University of Cambridge, Cambridge, UK

^d^INSERM, Centre for Research in Epidemiology & Public Health, Hôpital Paul Brousse, Bâtiment, France.

Short title: Adiposity and Aortic Stiffening

1 table and 2 figures

**Corresponding author**

Eric Brunner

UCL Research Department of Epidemiology and Public Health, 1-19 Torrington Place, London WC1E 6BT, UK

Tel: +44(0) 20 7679 1689

Fax: +44(0) 20 7419 6732

Email: [e.brunner@ucl.ac.uk](mailto:e.brunner@ucl.ac.uk)

**Methods**

*Aortic pulse wave velocity*

With the participant supine, blood pressure was measured twice after 10 minutes rest. From the supine systolic (SBP) and diastolic blood pressure (DBP), mean blood pressure (MAP) in mm Hg was calculated as: DBP + 0.33(SBP-DBP). PWV was assessed between carotid and femoral sites using applanation tonometry (SphygmoCor, Atcor Medical, Australia).^1^ Path length was determined with tape measure by subtracting carotid-sternal notch distance from femoral-sternal notch distance. In each participant, PWV was measured twice and if the difference between the two measurements was >0.5 m/s, a third measurement was taken. The average of measurements was used in analysis. PWV measurements were repeated within 30 days in 125 participants in 2008-09 and 114 participants in 2012-13 to assess short-term reproducibility. Median intra-individual difference in PWV was respectively 0.83 m/s (interquartile range 0.43-1.40) and 0.89 m/s (interquartile range 0.41-1.47).

*Vascular disease, diabetes and anti-hypertensive medication*

Prevalent vascular disease status (myocardial infarction and/or stroke) at the 2008-09 assessment was determined using self-report doctor diagnosis and hospitalization with verification from medical records where available. Prevalent diabetes was determined by oral glucose tolerance test, self-report doctor diagnosis and/or medication.^2^

*Anthropometry and other covariates*

Risk factors were measured in 2003-04 and 2008-09 to provide mean exposure in the 5 years before baseline PWV assessment in 2008-09. Weight, height, and waist and hip circumferences were measured using standard protocols.^3^ Fat mass was estimated with the Tanita TBF-300 body composition analyser.^4^ Serum, fluoride plasma and EDTA blood was collected after overnight fast or ≥4 hours after a fat-free breakfast for participants presenting in the afternoon. Serum total cholesterol, high density lipoprotein cholesterol (HDL), triglycerides, plasma glucose, serum C-reactive protein (CRP) and interleukin-6 (IL-6) were measured.

*Statistical analysis*

Distributions of adiposity measures were categorised in sex-specific thirds and standardised units. Linear mixed models were used to estimate the relation of adiposity with PWV in 2008-09 and change in PWV between 2008-09 and 2012-13. These models use all available PWV data and account for correlation between repeated measures within individuals. We fitted the intercept and slope with time as random effects for individual differences in PWV at baseline and rate of change over follow-up. The main effect for adiposity estimates the effect on PWV at baseline (2008-09) while the adiposityXtime interaction term estimates the effect of adiposity on change in PWV between 2008-09 and 2012-13 as a 5-year change.

Models were adjusted for age, sex, ethnic group and MAP at the time of PWV measurement. Change in PWV by third of each adiposity measure and per 1SD increment in adiposity was estimated. PWV changes were estimated separately among those who were metabolically healthy and unhealthy according to ATP-III criteria excluding waist circumference.^5^ Further models additionally adjusted for: (i) chronic disease and antihypertensive medication, (ii) heart rate, (iii) serum triglycerides, HDL, fasting glucose and haemoglobin A1c, (iv) CRP and IL-6 and (v) all factors. Main analyses using logarithmically transformed PWV, in place of PWV, produced similar strength of associations with adiposity. We conducted a sensitivity analysis to examine potential bias arising from change in central adiposity over the follow-up period.^6^ We calculated path length at follow-up adjusted for the association between change in waist circumference and change in measured aortic path length. Adjustment was based on a sex-specific regression model of change in path length against change in waist circumference. The path length was adjusted to remove the effect of change in waist circumference on path length in each individual. We used data from a published meta-analysis ^7^ to estimate change in CVD risk resulting from increased log PWV due to a 1SD higher BMI and expressed this change according to Wormser^8^ to estimate the proportion of this increase mediated through PWV.

*References*

1. Wilkinson IB, Fuchs SA, Jansen IM, [Spratt JC](http://www.ncbi.nlm.nih.gov/pubmed/?term=Spratt%20JC%5BAuthor%5D&cauthor=true&cauthor_uid=9886900), [Murray GD](http://www.ncbi.nlm.nih.gov/pubmed/?term=Murray%20GD%5BAuthor%5D&cauthor=true&cauthor_uid=9886900), [Cockcroft JR](http://www.ncbi.nlm.nih.gov/pubmed/?term=Cockcroft%20JR%5BAuthor%5D&cauthor=true&cauthor_uid=9886900), [Webb DJ](http://www.ncbi.nlm.nih.gov/pubmed/?term=Webb%20DJ%5BAuthor%5D&cauthor=true&cauthor_uid=9886900). Reproducibility of pulse wave velocity and augmentation index measured by pulse wave analysis. *J Hypertens.*1998;16:2079-2084.

2. Brunner EJ, Shipley MJ, Marmot MG, Kivimaki M, Witte DR. Do the Joint British Society (JBS2) guidelines on prevention of cardiovascular disease with respect to plasma glucose improve risk stratification in the general population? Prospective cohort study. *Diabet Med.* 2010;27:550-555.

3. Brunner EJ, Marmot MG, Nanchahal K, [Shipley MJ](http://www.ncbi.nlm.nih.gov/pubmed/?term=Shipley%20MJ%5BAuthor%5D&cauthor=true&cauthor_uid=9389428), [Stansfeld SA](http://www.ncbi.nlm.nih.gov/pubmed/?term=Stansfeld%20SA%5BAuthor%5D&cauthor=true&cauthor_uid=9389428), [Juneja M](http://www.ncbi.nlm.nih.gov/pubmed/?term=Juneja%20M%5BAuthor%5D&cauthor=true&cauthor_uid=9389428), [Alberti KG](http://www.ncbi.nlm.nih.gov/pubmed/?term=Alberti%20KG%5BAuthor%5D&cauthor=true&cauthor_uid=9389428). Social inequality in coronary risk: central obesity and the metabolic syndrome. Evidence from the Whitehall II study. *Diabetologia.* 1997;40:1341-1349.

4. Nunez C, Gallagher D, Visser M, Pi-Sunyer F, Wang Z, Heymsfield SB. Bioimpedance analysis: evaluation of leg-to-leg system based on pressure contact footpad electrodes. *Med Sci Sports Exerc.* 1997;29:524-531.

5. Executive Summary of The Third Report of The National Cholesterol Education Program (NCEP) Expert Panel on Detection, Evaluation, And Treatment of High Blood Cholesterol In Adults (Adult Treatment Panel III). *JAMA.* 2001;285:2486-2497.

6. Canepa M, AlGhatrif M, Pestelli G Kankaria R, Makrogiannis S, Strait JB, Brunelli C, Lakatta EG, Ferrucci L. Impact of central obesity on the estimation of carotid-femoral pulse wave velocity. *Am J Hypertens.* 2014;27:1209-1217.

7. Ben-Shlomo Y, Spears M, Boustred C et al. Aortic pulse wave velocity improves cardiovascular event prediction: an individual participant meta-analysis of prospective observational data from 17,635 subjects. *J Am Coll Cardiol.* 2014;63:636-646.

8. Wormser D, Kaptoge S, Di AE et al. Separate and combined associations of body-mass index and abdominal adiposity with cardiovascular disease: collaborative analysis of 58 prospective studies. *Lancet.* 2011;377:1085-1095.

Table S1. Characteristics of 5172 participants included in the analyses

| Characteristic | Men (N=3789) | |  | Women (N=1383) | |
| --- | --- | --- | --- | --- | --- |
|  | Mean (SD) | % (N) |  | Mean (SD) | % (N) |
|  |  |  |  |  |  |
| Age (2008-09), y | 65.5 (5.8) |  |  | 66.4 (5.8) |  |
|  |  |  |  |  |  |
| Age group (2008-09): 55 – 59 |  | 20.0 (759) |  |  | 21.6 (299) |
| 60 – 64 |  | 32.9 (1245) |  |  | 30.2 (418) |
| 65 – 69 |  | 21.3 (806) |  |  | 22.7 (314) |
| 70+ |  | 25.8 (979) |  |  | 25.5 (352) |
|  |  |  |  |  |  |
| Ethnic group: White |  | 94.1 (3566) |  |  | 87.4 (1209) |
| South Asian |  | 3.9 (149) |  |  | 5.9 (81) |
| Black |  | 1.4 (54) |  |  | 5.4 (74) |
| Other |  | 0.5 (20) |  |  | 1.4 (19) |
|  |  |  |  |  |  |
| Chronic disease (2008-09) |  | 16.0 (606) |  |  | 13.7 (190) |
|  |  |  |  |  |  |
| Anti-hypertensive medication (2008-09) |  | 34.5 (1307) |  |  | 31.5 (435) |
|  |  |  |  |  |  |
| Mean body mass index,* kg/m^2^ | 26.3 (3.5) |  |  | 26.6 (4.9) |  |
|  |  |  |  |  |  |
| Mean waist circumference,* m | 0.939 (0.100) |  |  | 0.834 (0.118) |  |
|  |  |  |  |  |  |
| Mean waist-hip ratio* | 0.940 (0.061) |  |  | 0.816 (0.065) |  |
|  |  |  |  |  |  |
| Mean fat mass percentage* | 23.9 (5.2) |  |  | 35.7 (6.6) |  |
|  |  |  |  |  |  |
| Mean HDL cholesterol, *,† mmol/L | 1.50 (0.38) |  |  | 1.86 (0.45) |  |
|  |  |  |  |  |  |
| Mean triglyceride,*,† mmol/L | 1.19 (0.47) |  |  | 1.04 (0.42) |  |
|  |  |  |  |  |  |
| Mean fasting glucose,*,† mmol/L | 5.36 (0.15) |  |  | 5.14 (0.14) |  |
|  |  |  |  |  |  |
| Mean HBA1c,*,† mmol/L | 5.49 (0.09) |  |  | 5.53 (0.10) |  |
|  |  |  |  |  |  |
| CRP (2003-04),† mg/L | 1.17 (1.06) |  |  | 1.41 (1.14) |  |
|  |  |  |  |  |  |
| IL6 (2003-04),† mg/L | 1.86 (0.57) |  |  | 1.73 (0.60) |  |
|  |  |  |  |  |  |
| Heart rate (2008-09), bpm | 66.1 (12.0) |  |  | 67.8 (10.8) |  |
|  |  |  |  |  |  |
| Pulse wave velocity (2008-09),‡ m/s | 8.54 (2.02) |  |  | 8.25 (2.02) |  |
|  |  |  |  |  |  |
| Mean arterial pressure (2008-09), mmHg | 90.5 (10.3) |  |  | 87.2 (11.1) |  |
|  |  |  |  |  |  |
| Pulse wave velocity (2012-13),§ m/s | 9.27 (2.54) |  |  | 8.88 (2.43) |  |
|  |  |  |  |  |  |
| Mean arterial pressure (2012-13), mmHg | 94.9 (10.6) |  |  | 91.2 (11.2) |  |
|  |  |  |  |  |  |

* From assessments in 2003-04 and 2008-09

† Geometric mean and SD logged values

‡ n=4328 (3223 men, 1105 women)

§ n=4316 (3202 men, 1114 women)

Table S2. Association between demographic and health factors and pulse wave velocity at baseline (2008-2009) and 5-year change in pulse wave velocity

| Characteristic | Pulse wave velocity at baseline  (2008-09) | | |  | Change in Pulse wave velocity  (per 5 years) | | |
| --- | --- | --- | --- | --- | --- | --- | --- |
|  | Mean | Difference (95% CI) | P-value |  | Mean | Increase (95% CI) | P-value |
|  |  |  |  |  |  |  |  |
| Age-group * (2008-09) |  |  |  |  |  |  |  |
| 55 – 59 | 7.59 | Ref | - |  | 0.18 | Ref | - |
| 60 – 64 | 8.13 | 0.55 (0.40, 0.69) | <0.001 |  | 0.36 | 0.18 (-0.01, 0.37) | 0.06 |
| 65 – 69 | 8.65 | 1.06 (0.91, 1.22) | <0.001 |  | 0.80 | 0.62 (0.41, 0.82) | <0.001 |
| 70+ | 9.72 | 2.14 (1.99, 2.29) | <0.001 |  | 0.99 | 0.81 (0.61, 1.01) | <0.001 |
|  |  |  |  |  |  |  |  |
| Sex† | |  |  |  |  |  |  |
| Men | 8.49 | Ref | - |  | 0.58 | Ref | - |
| Women | 8.44 | -0.05 (-0.17, 0.06) | 0.37 |  | 0.49 | -0.09 (-0.24, 0.07) | 0.27 |
|  |  |  |  |  |  |  |  |
| Ethnic group‡ |  |  |  |  |  |  |  |
| White | 8.44 | Ref | - |  | 0.55 | Ref | - |
| South Asian | 9.02 | 0.58 (0.33, 0.82) | <0.001 |  | 0.65 | 0.10 (-0.24, 0.43) | 0.58 |
| Black | 8.93 | 0.48 (0.15, 0.81) | 0.004 |  | 0.79 | 0.24 (-0.22, 0.69) | 0.31 |
| Other | 8.20 | -0.24 (-0.81, 0.32) | 0.40 |  | -0.02 | -0.57 (-1.34, 0.21) | 0.15 |
|  |  |  |  |  |  |  |  |
| Chronic disease§ (2008-09) |  |  |  |  |  |  |  |
| No | 8.38 | Ref | - |  | 0.48 | Ref | - |
| Yes | 9.11 | 0.73 (0.57, 0.89) | <0.001 |  | 0.81 | 0.33 (0.13, 0.52) | 0.001 |
|  |  |  |  |  |  |  |  |
| Anti-hypertensive medication§ (2008-09) |  |  |  |  |  |  |  |
| No | 8.35 | Ref | - |  | 0.45 | Ref | - |
| Yes | 8.76 | 0.40 (0.28, 0.52) | <0.001 |  | 0.69 | 0.23 (0.09, 0.38) | 0.002 |
|  |  |  |  |  |  |  |  |

* Pulse wave velocity estimates are adjusted for mean arterial pressure at the time of the pulse wave velocity measurement. Means are shown adjusted to a mean arterial pressure of 90mmHg.

† Pulse wave velocity estimates are adjusted for age, and mean arterial pressure at the time of the pulse wave velocity measurement. Means are shown adjusted to age 65 with a mean arterial pressure of 90mmHg.

‡ Pulse wave velocity estimates are adjusted for age, sex and mean arterial pressure at the time of the pulse wave velocity measurement. Means are shown adjusted to males, aged 65 with a mean arterial pressure of 90mmHg.

§ Pulse wave velocity estimates are adjusted for age, sex, ethnic group and mean arterial pressure at the time of the pulse wave velocity measurement. Means are shown adjusted to white males, aged 65 with a mean arterial pressure of 90mmHg.

Table S3. Association of mean* anthropometric measures with pulse wave velocity at baseline (2008-2009) and 5-year change in pulse wave velocity

| Anthropometric measure | Pulse wave velocity at baseline  (2008-09) | | |  | Change in Pulse wave velocity  (per 5 years) | | |
| --- | --- | --- | --- | --- | --- | --- | --- |
|  | Mean† | Difference‡ (95% CI) | P-value |  | Mean† | Increase‡ (95% CI) | P-value |
|  |  |  |  |  |  |  |  |
| Mean body mass index* | | |  |  |  |  |  |
| Lowest third | 8.26 | Ref | - |  | 0.42 | Ref | - |
| Middle third | 8.46 | 0.21 (0.09, 0.33) | <0.001 |  | 0.53 | 0.11 (-0.05, 0.27) | 0.17 |
| Highest third | 8.71 | 0.45 (0.32, 0.58) | <0.001 |  | 0.90 | 0.48 (0.32, 0.65) | <0.001 |
|  |  |  |  |  |  |  |  |
| Per 1SD higher BMI |  | 0.20 (0.14, 0.26) | <0.001 |  |  | 0.21 (0.13, 0.29) | <0.001 |
|  |  |  |  |  |  |  |  |
|  |  |  |  |  |  |  |  |
| Mean waist circumference* | | |  |  |  |  |  |
| Lowest third | 8.14 | Ref | - |  | 0.44 | Ref | - |
| Middle third | 8.53 | 0.39 (0.27, 0.51) | <0.001 |  | 0.46 | 0.02 (-0.14, 0.17) | 0.82 |
| Highest third | 8.77 | 0.63 (0.50, 0.75) | <0.001 |  | 0.96 | 0.52 (0.35, 0.69) | <0.001 |
|  |  |  |  |  |  |  |  |
| Per 1SD higher waist |  | 0.29 (0.24, 0.35) | <0.001 |  |  | 0.23 (0.16, 0.31) | <0.001 |
|  |  |  |  |  |  |  |  |
|  |  |  |  |  |  |  |  |
| Mean waist-hip ratio* | | |  |  |  |  |  |
| Lowest third | 8.14 | Ref | - |  | 0.42 | Ref | - |
| Middle third | 8.46 | 0.33 (0.21, 0.45) | <0.001 |  | 0.51 | 0.10 (-0.06, 0.25) | 0.23 |
| Highest third | 8.86 | 0.72 (0.60, 0.85) | <0.001 |  | 0.91 | 0.49 (0.33, 0.66) | <0.001 |
|  |  |  |  |  |  |  |  |
| Per 1SD higher WHR |  | 0.34 (0.29, 0.40) | <0.001 |  |  | 0.20 (0.13, 0.28) | <0.001 |
|  |  |  |  |  |  |  |  |
|  |  |  |  |  |  |  |  |
| Mean fat mass percent* | | |  |  |  |  |  |
| Lowest third | 8.20 | Ref | - |  | 0.42 | Ref | - |
| Middle third | 8.48 | 0.28 (0.16, 0.40) | <0.001 |  | 0.55 | 0.13 (-0.02, 0.29) | 0.10 |
| Highest third | 8.72 | 0.52 (0.39, 0.64) | <0.001 |  | 0.85 | 0.43 (0.26, 0.60) | <0.001 |
|  |  |  |  |  |  |  |  |
| Per 1SD higher FMP |  | 0.22 (0.16, 0.27) | <0.001 |  |  | 0.19 (0.12, 0.26) | <0.001 |

Analyses for BMI, waist circumference and waist hip ratio are based on 8636 pulse wave velocity observations in 5172 participants and those for fat mass percent based on 8488 observations in 5031 participants.

* From assessments in 2003-04 and 2008-09

† Means are adjusted for age, sex, ethnic group and mean arterial pressure at the time of the pulse wave velocity measurement and are shown adjusted to white men at age 65 with a mean arterial pressure of 90mmHg.

‡ Cross-sectional differences or longitudinal increases are adjusted for age, ethnic group and mean arterial pressure at the time of the pulse wave velocity

Table S4. Association of mean* anthropometric measures with pulse wave velocity at baseline (2008-2009) and adjusted† 5-year change in pulse wave velocity. PWV path length adjusted for change in waist circumference between 2008-09 and 2012-13.

| Anthropometric measure | Model adjustments | Pulse wave velocity at baseline  (2008-09) | |  | Change in Pulse wave velocity  (per 5 years) | |
| --- | --- | --- | --- | --- | --- | --- |
|  |  | Difference‡ (95% CI) | P-value |  | Increase‡ (95% CI) | P-value |
|  |  |  |  |  |  |  |
| Body mass index | Model 1§ | 0.20 (0.14, 0.26) | <0.001 |  | 0.22 (0.14, 0.30) | <0.001 |
|  | Model 1 + chronic disease, antihypertensive medication | 0.14 (0.09, 0.20) | <0.001 |  | 0.20 (0.12, 0.28) | <0.001 |
|  | Model 1 + heart rate | 0.14 (0.09, 0.20) | <0.001 |  | 0.22 (0.14, 0.30) | <0.001 |
|  | Model 1 + triglyceride, HDL, fasting glucose, HbA1_c_ | 0.06 (0.00, 0.12) | 0.06 |  | 0.19 (0.11, 0.28) | <0.001 |
|  | Model 1 + CRP, IL6 | 0.12 (0.05, 0.19) | <0.001 |  | 0.18 (0.09, 0.26) | <0.001 |
|  | Model 1 + All factors | -0.03 (-0.09, 0.04) | 0.46 |  | 0.15 (0.06, 0.25) | 0.001 |
|  |  |  |  |  |  |  |
| Waist circumference | Model 1§ | 0.30 (0.24, 0.35) | <0.001 |  | 0.24 (0.17, 0.32) | <0.001 |
|  | Model 1 + chronic disease, antihypertensive medication | 0.24 (0.19, 0.30) | <0.001 |  | 0.22 (0.15, 0.30) | <0.001 |
|  | Model 1 + heart rate | 0.22 (0.17, 0.28) | <0.001 |  | 0.25 (0.17, 0.33) | <0.001 |
|  | Model 1 + triglyceride, HDL, fasting glucose, HbA1_c_ | 0.16 (0.10, 0.23) | <0.001 |  | 0.23 (0.14, 0.31) | <0.001 |
|  | Model 1 + CRP, IL6 | 0.24 (0.17, 0.31) | <0.001 |  | 0.20 (0.11, 0.29) | <0.001 |
|  | Model 1 + All factors | 0.08 (0.01, 0.15) | 0.03 |  | 0.19 (0.10, 0.29) | <0.001 |
|  |  |  |  |  |  |  |
| Waist-hip ratio | Model 1§ | 0.34 (0.29, 0.40) | <0.001 |  | 0.21 (0.14, 0.28) | <0.001 |
|  | Model 1 + chronic disease, antihypertensive medication | 0.29 (0.24, 0.35) | <0.001 |  | 0.20 (0.12, 0.27) | <0.001 |
|  | Model 1 + heart rate | 0.27 (0.21, 0.32) | <0.001 |  | 0.22 (0.15, 0.29) | <0.001 |
|  | Model 1 + triglyceride, HDL, fasting glucose, HbA1_c_ | 0.23 (0.17, 0.29) | <0.001 |  | 0.19 (0.11, 0.27) | <0.001 |
|  | Model 1 + CRP, IL6 | 0.31 (0.25, 0.37) | <0.001 |  | 0.17 (0.09, 0.25) | <0.001 |
|  | Model 1 + All factors | 0.16 (0.09, 0.22) | <0.001 |  | 0.17 (0.08, 0.25) | <0.001 |
|  |  |  |  |  |  |  |
| Fat mass percent | Model 1§ | 0.22 (0.16, 0.27) | <0.001 |  | 0.19 (0.12, 0.27) | <0.001 |
|  | Model 1 + chronic disease, antihypertensive medication | 0.17 (0.12, 0.23) | <0.001 |  | 0.18 (0.11, 0.25) | <0.001 |
|  | Model 1 + heart rate | 0.15 (0.10, 0.21) | <0.001 |  | 0.19 (0.12, 0.27) | <0.001 |
|  | Model 1 + triglyceride, HDL, fasting glucose, HbA1_c_ | 0.10 (0.04, 0.15) | <0.001 |  | 0.17 (0.09, 0.25) | <0.001 |
|  | Model 1 + CRP, IL6 | 0.15 (0.09, 0.21) | <0.001 |  | 0.16 (0.08, 0.24) | <0.001 |
|  | Model 1 + All factors | 0.02 (-0.05, 0.08) | 0.57 |  | 0.14 (0.06, 0.23) | 0.001 |

Analyses for BMI, waist circumference and waist hip ratio are based on 8636 pulse wave velocity observations in 5172 participants and those for fat mass percent based on 8488 observations in 5031 participants. Because of missing values for CRP and IL6, models that include CRP and IL6 are based on 10% fewer observations

* From assessments in 2003-04 and 2008-09

† Pulse wave velocity at 2012-2013 calculated using path length that has been adjusted for change in waist circumference between 2008-09 and 2012-13

‡ Cross-sectional difference or longitudinal increase in the outcome associated with a 1 standard deviation higher value in each anthropometric measure

§ Model 1 is adjusted for age, sex, ethnic group and mean arterial pressure at the time of the pulse wave velocity measurement.

Table S5. Association of mean* anthropometric measures with pulse wave velocity at baseline (2008-2009) and adjusted† 5-year change in pulse wave velocity according to the path length used to calculate pulse wave velocity at follow-up.

| Anthropometric measure | Path length used in calculation of pulse wave velocity at follow-up (2012-13) | Pulse wave velocity at baseline  (2008-09) | |  | Change in Pulse wave velocity  (per 5 years) | |
| --- | --- | --- | --- | --- | --- | --- |
|  |  | Difference‡ (95% CI) | P-value |  | Increase‡ (95% CI) | P-value |
|  |  |  |  |  |  |  |
| Body mass | Path length at follow-up (from Model 1 in Table 2) | 0.20 (0.14, 0.26) | <0.001 |  | 0.22 (0.14, 0.30) | <0.001 |
| Index | Path length at baseline used when measured | 0.20 (0.14, 0.25) | <0.001 |  | 0.16 (0.08, 0.23) | <0.001 |
|  | Path length at follow-up (restricted sample§) | 0.20 (0.15, 0.26) | <0.001 |  | 0.22 (0.14, 0.30) | <0.001 |
|  | Path length at baseline (restricted sample) | 0.21 (0.15, 0.27) | <0.001 |  | 0.18 (0.10, 0.26) | <0.001 |
|  |  |  |  |  |  |  |
| Waist | Path length at follow-up (from Model 1 in Table 2) | 0.29 (0.24, 0.35) | <0.001 |  | 0.23 (0.16, 0.31) | <0.001 |
| Circumference | Path length at baseline | 0.29 (0.23, 0.35) | <0.001 |  | 0.17 (0.09, 0.25) | <0.001 |
|  | Path length at follow-up (restricted sample) | 0.29 (0.24, 0.35) | <0.001 |  | 0.23 (0.15, 0.31) | <0.001 |
|  | Path length at baseline (restricted sample) | 0.30 (0.24, 0.36) | <0.001 |  | 0.19 (0.11, 0.27) | <0.001 |
|  |  |  |  |  |  |  |
| Waist-hip ratio | Path length at follow-up (from Model 1 in Table 2) | 0.34 (0.29, 0.40) | <0.001 |  | 0.20 (0.13, 0.28) | <0.001 |
|  | Path length at baseline used when measured | 0.34 (0.29, 0.39) | <0.001 |  | 0.18 (0.11, 0.25) | <0.001 |
|  | Path length at follow-up (restricted sample) | 0.34 (0.29, 0.40) | <0.001 |  | 0.20 (0.12, 0.27) | <0.001 |
|  | Path length at baseline (restricted sample) | 0.34 (0.29, 0.40) | <0.001 |  | 0.19 (0.11, 0.26) | <0.001 |
|  |  |  |  |  |  |  |
| Fat mass | Path length at follow-up (from Model 1 in Table 2) | 0.22 (0.16, 0.27) | <0.001 |  | 0.19 (0.12, 0.26) | <0.001 |
| Percent | Path length at baseline used when measured | 0.22 (0.16, 0.27) | <0.001 |  | 0.14 (0.07, 0.21) | <0.001 |
|  | Path length at follow-up (restricted sample) | 0.22 (0.17, 0.28) | <0.001 |  | 0.19 (0.12, 0.27) | <0.001 |
|  | Path length at baseline (restricted sample) | 0.23 (0.17, 0.28) | <0.001 |  | 0.16 (0.08, 0.23) | <0.001 |
|  |  |  |  |  |  |  |

* From assessments in 2003-04 and 2008-09

† Pulse wave velocity at 2012-2013 calculated using path length that has been adjusted for change in waist circumference between 2008-09 and 2012-13

‡ Cross-sectional difference or longitudinal increase in the outcome associated with a 1 standard deviation higher value in each anthropometric measure. Estimates are adjusted for age, sex, ethnic group and mean arterial pressure at the time of the pulse wave velocity measurement.

§ Restricted sample excludes 844 individuals who did not have pulse wave velocity measured at baseline

Table S6. Association of mean* adiposity measures with 5-year change in pulse wave velocity adjusted for the longitudinal effects of risk factors measured at baseline (2008-2009) and the concurrent effects of adiposity and risk factors at the second PWV assessment (2012-2013).

|  | Change in Pulse wave velocity (per 5 years) | |
| --- | --- | --- |
| Anthropometric measure | Increase† (95% CI) | Increase† (95% CI) |
|  |  |  |
|  | Model 1‡ | Model 2§ |
|  |  |  |
| Mean* body mass index | 0.16 (0.07, 0.26) | 0.15 (0.05, 0.24) |
| Mean* waist circumference | 0.19 (0.09, 0.29) | 0.16 (0.07, 0.26) |
| Mean* waist-hip ratio | 0.17 (0.08, 0.26) | 0.13 (0.05, 0.22) |
| Mean* fat mass percent | 0.17 (0.08, 0.26) | 0.15 (0.06, 0.24) |

* From assessments in 2003-04 and 2008-09 using linear mixed model, as in Table 2

† Change in pulse wave velocity (per 5 years) per 1 SD higher level of each adiposity measure

‡ Model 1: adjusted for age, sex, ethnic group and mean arterial pressure at the time of the pulse wave velocity, chronic disease, antihypertensive medication, the longitudinal effects of triglyceride, HDL, fasting glucose, HbA1_c_, CRP, IL6 and heart rate measured at baseline (2008-2009) and the concurrent effects of adiposity at the second PWV assessment

§ Model2: adjusted as in Model 1 + the concurrent effects of triglyceride, HDL, fasting glucose, HbA1_c_ and heart rate at the second PWV assessment

Table S7. Association of mean* adiposity measures with 5-year change in pulse wave velocity stratified by change in adiposity between baseline (2008-2009) and follow-up (2012-2013).

| Anthropometric measure | Change in adiposity between baseline (2008-2009) and follow-up (2012-2013) | | P-value for difference |
| --- | --- | --- | --- |
|  | ≤ median† | > median |  |
|  | Increase‡ (95% CI) | Increase‡ (95% CI) |  |
|  |  |  |  |
| Mean* body mass index | 0.12 (0.00, 0.24) | 0.17 (0.05, 0.29) | 0.52 |
| Mean* waist circumference | 0.11 (-0.01, 0.23) | 0.25 (0.12, 0.37) | 0.08 |
| Mean* waist-hip ratio | 0.15 (0.04, 0.26) | 0.15 (0.04, 0.27) | 0.99 |
| Mean* fat mass percent | 0.13 (0.01, 0.24) | 0.17 (0.05, 0.28) | 0.57 |

* From assessments in 2003-04 and 2008-09 using linear mixed model, as in Table 2, with stratification by sex-specific median change in adiposity variable between 2008-2009 and 2012-2013.

† Median cut-points for change in adiposity, in men and women respectively, are:- 0.06 and 0.04 Kgm^-2^ for BMI; 1.7 and 2.1 cm for waist circumference; 0.014 and 0.018 for waist-hip ratio; -0.2 and -0.6 for fat mass percent

‡ Change in pulse wave velocity (per 5 years) per 1 SD higher level of each adiposity measure, adjusted for age, sex, ethnic group and mean arterial pressure at the time of the pulse wave velocity, chronic disease, antihypertensive medication, triglyceride, HDL, fasting glucose, HbA1_c_, CRP, IL6 and heart rate

Table S8. Association of mean* anthropometric measures with pulse wave velocity at baseline (2008-2009) and 5-year change in pulse wave velocity by sub-group

| Anthropometric measure | Sub-group |  | Pulse wave velocity at baseline (2008-09) | |  | Change in Pulse wave velocity (per 5 years) | |
| --- | --- | --- | --- | --- | --- | --- | --- |
|  |  | Person-observations | Difference† (95% CI) | P-value for difference between sub-groups |  | Increase† (95% CI) | P-value for difference between sub-groups |
|  |  |  |  |  |  |  |  |
| Body mass index | Men | 6419 | 0.23 (0.16, 0.30) | 0.11 |  | 0.23 (0.14, 0.32) | 0.31 |
|  | Women | 2217 | 0.11 (-0.01, 0.22) |  |  | 0.14 (-0.01, 0.29) |  |
|  |  |  |  |  |  |  |  |
|  | Normotensive | 5031 | 0.18 (0.11, 0.25) | 0.41 |  | 0.06 (-0.03, 0.15) | 0.001 |
|  | Hypertensive | 3605 | 0.14 (0.04, 0.24) |  |  | 0.34 (0.20, 0.48) |  |
|  |  |  |  |  |  |  |  |
|  | Non-diabetic | 7542 | 0.18 (0.11, 0.24) | 0.15 |  | 0.20 (0.12, 0.28) | 0.97 |
|  | Diabetic | 941 | 0.10 (-0.10, 0.29) |  |  | 0.16 (-0.13, 0.44) |  |
|  |  |  |  |  |  |  |  |
| Waist circumference | Men | 6419 | 0.32 (0.25, 0.39) | 0.18 |  | 0.25 (0.16, 0.34) | 0.30 |
|  | Women | 2217 | 0.22 (0.11, 0.33) |  |  | 0.16 (0.01, 0.30) |  |
|  |  |  |  |  |  |  |  |
|  | Normotensive | 5031 | 0.27 (0.20, 0.35) | 0.86 |  | 0.11 (0.02, 0.19) | 0.005 |
|  | Hypertensive | 3605 | 0.26 (0.16, 0.35) |  |  | 0.33 (0.20, 0.47) |  |
|  |  |  |  |  |  |  |  |
|  | Non-diabetic | 7542 | 0.26 (0.20, 0.32) | 0.32 |  | 0.22 (0.14, 0.30) | 0.84 |
|  | Diabetic | 941 | 0.22 (0.03, 0.42) |  |  | 0.15 (-0.14, 0.44) |  |
|  |  |  |  |  |  |  |  |
| Waist-hip ratio | Men | 6419 | 0.35 (0.29, 0.41) | 0.94 |  | 0.23 (0.14, 0.31) | 0.23 |
|  | Women | 2217 | 0.33 (0.23, 0.44) |  |  | 0.12 (-0.01, 0.26) |  |
|  |  |  |  |  |  |  |  |
|  | Normotensive | 5031 | 0.30 (0.24, 0.36) | 0.29 |  | 0.15 (0.07, 0.24) | 0.46 |
|  | Hypertensive | 3605 | 0.33 (0.24, 0.42) |  |  | 0.21 (0.09, 0.34) |  |
|  |  |  |  |  |  |  |  |
|  | Non-diabetic | 7542 | 0.29 (0.24, 0.35) | 0.55 |  | 0.18 (0.11, 0.26) | 0.83 |
|  | Diabetic | 941 | 0.37 (0.18, 0.56) |  |  | 0.14 (-0.14, 0.42) |  |
|  |  |  |  |  |  |  |  |
| Fat mass  percent | Men | 6329 | 0.24 (0.17, 0.30) | 0.29 |  | 0.22 (0.14, 0.31) | 0.10 |
|  | Women | 2159 | 0.16 (0.06, 0.26) |  |  | 0.09 (-0.04, 0.22) |  |
|  |  |  |  |  |  |  |  |
|  | Normotensive | 4975 | 0.19 (0.12, 0.25) | 0.63 |  | 0.10 (0.02, 0.18) | 0.04 |
|  | Hypertensive | 3513 | 0.20 (0.10, 0.30) |  |  | 0.26 (0.13, 0.40) |  |
|  |  |  |  |  |  |  |  |
|  | Non-diabetic | 7429 | 0.20 (0.14, 0.25) | 0.28 |  | 0.19 (0.12, 0.26) | 0.45 |
|  | Diabetic | 921 | 0.13 (-0.07, 0.34) |  |  | 0.08 (-0.21, 0.38) |  |

* From assessments in 2003-04 and 2008-09. Blood pressure and diabetes sub-groups defined by status in 2008-09.

† Cross-sectional difference or longitudinal increase in the outcome associated with 1 SD higher adiposity. Estimates are adjusted for age, sex, ethnic group and mean arterial pressure at the time of the pulse wave velocity measurement
